# Protection‐Free, Two‐step Synthesis of C5‐C Functionalized Pyrimidine Nucleosides

**DOI:** 10.1002/cpz1.984

**Published:** 2024-02-07

**Authors:** Karolina Podskoczyj, Anna Kuszczynska, Agnieszka Dziergowska, Grazyna Leszczynska

**Affiliations:** ^1^ Institute of Organic Chemistry, Faculty of Chemistry Lodz University of Technology Lodz Poland

**Keywords:** 5‐trifluoromethylcytidine, 5‐trifluoromethyluridine, C5‐modified pyrimidine nucleosides, CF_3_‐group transformations, gram‐scale chemical synthesis

## Abstract

A simple, reliable, and efficient method for the gram‐scale chemical synthesis of pyrimidine nucleosides functionalized with C5‐carboxyl, nitrile, ester, amide, or amidine, starting from unprotected uridine and cytidine, is described. The protocol involves the synthesis of 5‐trifluoromethyluridine and 5‐trifluoromethylcytidine with Langlois reagent (CF_3_SO_2_Na) in the presence of *tert*‐butyl hydroperoxide and subsequent transformation of the CF_3_ group to the C5‐C ‘carbon substituents’ under alkaline conditions. © 2024 The Author(s). *Current Protocols* published by Wiley Periodicals LLC.

**Basic Protocol 1**: Synthesis and characterization of 5‐trifluoromethyluridine (5‐CF_3_U) and 5‐trifluoromethylcytidine (5‐CF_3_C)

**Basic Protocol 2**: Conversion of 5‐CF_3_U and 5‐CF_3_C to several C5‐substituted ribonucleosides

## INTRODUCTION

5‐Substituted pyrimidine nucleosides belong to a widespread class of modified nucleosides identified in all types of cellular ribonucleic acids (RNAs; see Internet Resources, Modomics), particularly transfer RNAs (Boccaletto et al., 2022), affecting their stability, folding, protein recruitment, and translation (Duechler et al., 2016). In several cases, the lack of 5‐substituted uridines/cytidines at specific RNA positions e.g., tRNA wobble position (first anticodon letter), causes severe human diseases or pathogenic damages of protein biosynthesis (Torres et al., 2014; Van Haute et al., 2016). In the last decade, the regulatory role of 5‐substituted cytidines and uridines in gene expression have been discovered. The mechanisms of the modified nucleoside‐dependent control of gene expression involve the dynamic and reversible conversions of epigenetic mRNA modifications (Alagia & Gullerova, 2022), acceleration/deceleration of tRNA modifying processes (Dedon & Begley, 2014; Dumelin et al., 2012) or tRNA cleavage (Schaefer et al., 2010; Tuorto et al., 2012). The synthetically‐obtained RNA modified nucleosides are simplified models to study the structure‐function relationship by investigation of their physicochemical and structural properties and/or computational analysis (Leszczynska et al., 2020; Sochacka et al., 2017).

5‐Substituted pyrimidine nucleosides are also widely studied as potential pharmaceutical agents with antibacterial, antiviral, and antitumor properties (Harki et al., 2006; Johar et al., 2005; Kozak et al., 2020; Liu et al., 2008; Mekras et al., 1985). The presence of a C5‐substituent in uridine/cytidine nucleoside was found to improve important drug‐like properties, such as bioavailability, stability under cellular conditions, and therapeutic activity. Notably, the 5‐CF_3_‐dU nucleoside is an antiviral drug commercially known as trifluridine, approved by the Food and Drug Administration (FDA) for the treatment of epithelial keratitis caused by herpes simplex virus 1 and 2 (HSV‐1 and HSV‐2).

In this context, there is a high demand to develop simple and effective methods for obtaining natural and artificial C5‐substituted pyrimidine nucleosides, particularly those with a C5‐C bond, which is challenging to form. Several methods for introducing a ‘carbon substituent’ into the C5 position of pyrimidine ribonucleosides have been reported (Crouch & Eaton, 1994; Dai et al., 2011; Fu et al., 2010; Karino et al., 2001; Reese & Sanghvi, 1984). Most of these methods require additional ribose protection and a final step of deprotection, which makes them costly and time‐consuming. Recently, our group reported a straightforward and effective method for the direct trifluoromethylation of uridine and cytidine (Podskoczyj et al., 2023), utilizing a radical approach documented in the literature for the synthesis of CF_3_‐containing deoxynucleosides (Ito et al., 2017; Ji et al., 2011; Musumeci et al., 2013). Subsequently, 5‐trifluoromethylated uridine (5‐CF_3_U) and cytidine (5‐CF_3_C) were converted to the C5‐carboxyl, ester, cyano, amide, or amidine functionalized derivatives (Podskoczyj et al., 2023).

This article describes the two‐step approach to introduce several chemically diverse substituents at the C5 position of uridine and cytidine via CF_3_‐functionalized derivatives (Podskoczyj et al., 2023). Basic Protocol 1 describes the synthesis and purification of 5‐CF_3_U and 5‐CF_3_C. Basic Protocol 2 outlines the conversions of trifluoromethylated uridine and cytidine (**2a** and **2b**) using oxygen and nitrogen nucleophiles.

*CAUTION*: All reactions must be run in a suitable fume hood with efficient ventilation. The use of protective equipment, e.g., safety glasses, gloves, and laboratory coats, is recommended.

*NOTE*: All glassware and equipment should be well‐dried before starting the reaction.

## Basic Protocol 1 SYNTHESIS AND CHARACTERIZATION OF 5‐TRIFLUOROMETHYLURIDINE (5‐CF_3_U) AND 5‐TRIFLUOROMETHYLCYTIDINE (5‐CF_3_C)

**Figure 1:**
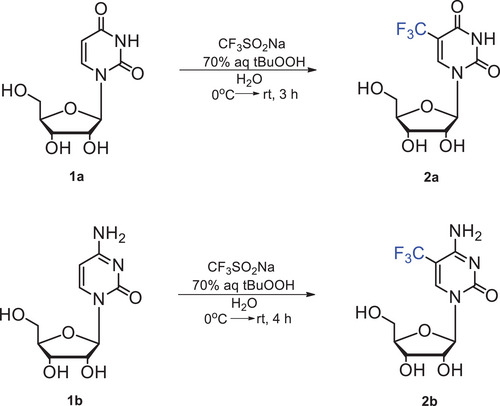
Synthesis of 5‐trifluoromethyluridine (5‐CF_3_U, **2a**) and 5‐trifluoromethylcytidine (5‐CF_3_C, **2b**).

The protocol describes a convenient and reliable method of 5‐CF_3_U (**2a**) and 5‐CF_3_C (**2b**) synthesis starting from commercially available uridine (U, **1a**) and cytidine (C, **1b**), respectively, as outlined in Figure 1. The procedure involves regioselective trifluoromethylation of **1a** and **1b** with sodium trifluoromethanesulfinate (Langlois reagent, CF_3_SO_2_Na) in the presence of *tert*‐butyl hydrogen peroxide as an oxidant (Ito et al., 2017; Ji et al., 2011; Musumeci et al., 2013). Trifluoromethylated compounds **2a** and **2b** were obtained in 81% and 72% yields, respectively.

### Materials

Uridine (**1a**, Sigma‐Aldrich, ≥99%, CAS: 58‐96‐8)
Cytidine (**1b**, Sigma‐Aldrich, ≥99%, CAS: 65‐46‐3)
H_2_O, distilled
Sodium trifluoromethanesulfinate (CF_3_SO_2_Na, Sigma‐Aldrich, ≥95.0% (T), CAS: 2926‐29‐6)
*tert*‐Butyl hydroperoxide solution (TBHP), 70% w/v in H_2_O (Sigma‐Aldrich, CAS: 75‐91‐2)
Chloroform (CHCl_3_)
Methanol (MeOH)
Anhydrous toluene

100‐ml and 50‐ml round‐bottom flasks
Magnetic stir bar
Glass stopper
Magnetic stirring plate
Ice‐water bath
Thin‐layer chromatography (TLC) plates, silica gel, 60 F_254_ (Supelco, cat. no. 105554)
UV lamp, 245 nm
Rotary evaporator
Vacuum pump
Filter funnel with sintered glass disc
Silica gel, 60‐Å pore size, 230 to 400 mesh particle size, 40‐ to 63‐µm particle size (Supelco, cat. no. 217441)
Glass columns for silica gel chromatography, 50‐mm width

Additional reagents and equipment for TLC (Meyers & Meyers, 2008) and column chromatography (Meyers, 2001)

*CAUTION: Tert*‐butyl hydroperoxide is a dangerous chemical that is highly reactive, flammable, and toxic. It is corrosive to skin and mucous membranes and causes respiratory distress when inhaled. Any operation involving this reagent should be performed with gloves under a well‐ventilated fume hood. Due to its reactivity and instability in contact with metal, non‐metallic syringes, glassware, or plastic labware (e.g., pipette tips) are preferred for handling TBHP.

1 Transfer 1.00 g (4.11 mmol) uridine/cytidine (**1a**/**1b**) into a 100‐ml round‐bottom flask equipped with a magnetic stir bar. Add 12 ml H_2_O and 1.92 g (12.33 mmol) sodium trifluoromethanesulfinate (CF_3_SO_2_Na). Seal the flask with a glass stopper.
2 Place the flask on a magnetic stirring plate and stir until a clear solution is formed.
3 Cool the mixture in an ice‐water bath.
4 Add 2.82 ml (20.55 mmol) of 70% *tert*‐butyl hydroperoxide in H_2_O dropwise at 0°C over 30 min.
  See the Caution after the materials list for handling tert‐butyl hydroperoxide.
5 Stir the mixture for 3 hr (substrate **1a**) or 4 hr (substrate **1b**) at room temperature. Check the reaction progress by TLC using 4:1 (v/v) CHCl_3_/MeOH as the eluent. Use a UV lamp (254 nm) to visualize the results.
  The conversion of uridine (**1a**) is nearly complete after 3 hr, and cytidine (**1b**) after 4 hr. In addition to the product and residual substrate, some trace impurities can be detected. Extending the reaction time or increasing the excess of reagents does not improve substrate consumption.
  The R_f_ of 5‐trifluoromethyluridine (**2a**) is 0.57.
  The R_f_ of 5‐trifluoromethylcytidine (**2b**) is 0.34.
6 Concentrate the contents of the round‐bottom flask on a rotary evaporator. Co‐evaporate the residue with anhydrous toluene (2 × 15 ml) using a rotary evaporator and a vacuum pump.
7 Add 20 ml methanol and mix the resulting suspension. Filter off the insoluble residue using a glass filter funnel. Rinse the residue with methanol (3 × 10 ml). Collect the filtrate in a 100‐ml round‐bottom flask and evaporate the mixture under reduced pressure.
  TLC control of the precipitate and methanol solution indicates that product **2a**/**2b** occurs only in the liquid phase.
8 Purify the crude compound **2a**/**2b** by silica gel column chromatography using a gradient of MeOH in CHCl_3_ as eluent (0% to 12% for compound **2a**, 0% to 18% for compound **2b**).
  Due to the poor solubility of 5‐CF_3_U and 5‐CF_3_C in CHCl_3_, the crude products are dry‐loaded onto the columns. Dissolve the sample in an appropriate solvent, e.g., methanol. Transfer it to a round‐bottom flask and add dry silica gel (∼10 to 20 times more than the sample by weight). Stir gently until the silica gel is completely suspended in the solution. Evaporate the solvent using a rotary evaporator until the silica is dry and free flowing. If it is still not powder, add more silica gel, and repeat the procedure. Pour the dry, compound‐adsorbing silica gel into the column and slowly add the solvent.
  The pure product should appear as a white solid (or slightly yellow). If the product is yellow‐orange and some artifacts are visible in the nuclear magnetic resonance spectroscopy (NMR) spectrum, repeat the purification by column chromatography.
9 Characterize **2a**/**2b** using ^1^H NMR, ^13^C NMR, ^19^F NMR, TLC, and high‐resolution mass spectrometry (HRMS).
  5‐Trifluoromethyluridine (**2a**): 81% yield (1.02 g) as a white solid. R_f_ = 0.57 (CHCl_3_/MeOH, 4:1, v/v). ^1^H NMR (700 MHz, D_2_O) δ (ppm): 8.77 (s, 1H, H6), 5.93 (d, 1H, J = 2.5 Hz, H1′), 4.38 (dd, 1H, J = 2.5 Hz, J = 5.0 Hz, H2′), 4.29 (dd, 1H, J = 5.0 Hz, J = 7.2 Hz, H3′), 4.20 (dt, 1H, J = 2.7 Hz, J = 7.2 Hz, H4′), 4.05 (dd, 1H, J = 2.5 Hz, J = 13.0 Hz, H5′), 3.89 (dd, 1H, J = 2.7 Hz, J = 13.0 Hz, H5″). ^13^C NMR (176 MHz, D_2_O) δ (ppm): 161.54 (C4), 150.68 (C2), 142.90 (q, J = 5.3 Hz, C6), 122.15 (q, J = 268.8 Hz, CF_3_), 104.36 (q, J = 33.3 Hz, C5), 90.39 (C1′), 83.76 (C4′), 74.34 (C2′), 68.26 (C3′), 59.35 (C5′). ^19^F NMR (376 MHz, D_2_O) δ: –63.26. HRMS (ESI) m/z calculated for C_10_H_10_N_2_O_6_F_3_ [M‐H]^‐^ 311.0490; found 311.0496.
  5‐Trifluoromethylcytidine (**2b**): 72% yield (0.92 g) as a white solid. R_f_ = 0.34 (CHCl_3_/MeOH, 4:1, v/v). ^1^H NMR (700 MHz, D_2_O) δ (ppm): 8.76 (s, 1H, H6), 5.90 (d, 1H, J = 2.1 Hz, H1′), 4.32 (dd, 1H, J = 2.1 Hz, J = 4.9 Hz, H2′), 4.25 (dd, 1H, J = 4.9 Hz, J = 8.0 Hz, H3′), 4.19 (dt, 1H, J = 2.8 Hz, J = 8.0 Hz, H4′), 4.07 (dd, 1H, J = 2.8 Hz, J = 13.3 Hz, H5′), 3.89 (dd, 1H, J = 2.8 Hz, J = 13.3 Hz, H5″). ^13^C NMR (176 MHz, D_2_O) δ (ppm): 161.30 (C4), 156.27 (C2), 143.43 (q, J = 6.2 Hz, C6), 122.8 (q, J = 269.5 Hz, CF_3_), 97.69 (q, J = 35.0 Hz, C5), 91.08 (C1′), 83.27 (C4′), 74.54 (C2′), 67.90 (C3′), 59.18 (C5′). ^19^F NMR (376 MHz, D_2_O) δ (ppm): –62.70. HRMS (ESI) m/z calculated for C_10_H_11_N_3_O_5_F_3_ [M‐H]^‐^ 310.0651; found 310.0660.

## Basic Protocol 2 CONVERSION OF 5‐CF_3_U and 5‐CF_3_C TO SEVERAL C5‐SUBSTITUTED RIBONUCLEOSIDES

**Figure 2:**
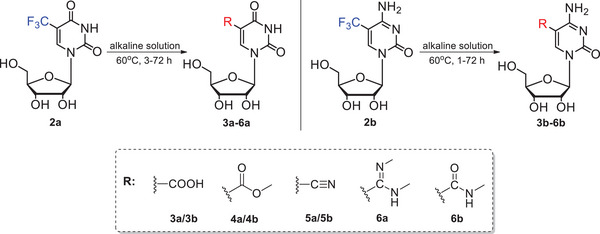
Transformations of 5‐CF_3_U (**2a**) and 5‐CF_3_C (**2b**).

The protocol describes the reactions of 5‐trifluoromethylated uridine (**2a**) and cytidine (**2b**) with nitrogen and oxygen nucleophiles, i.e., hydroxide and methoxy anions, ammonia, and methylamine, leading to the conversion of 5‐CF_3_ group into carboxyl, ester, cyano, amide, or amidine moieties, as outlined in Figure 2. CF_3_‐conversions were initially optimized on uridine **2a** as a substrate and then adopted to CF_3_‐cytidine (**2b**), and scale‐up, achieving conversion yields from 60% to 90%.

### Materials

5‐Trifluoromethyluridine (**2a**) and 5‐trifluoromethylcytidine (**2b**) (see Basic Protocol 1)
0.5 M aqueous sodium hydroxide (NaOH) (Current Protocols, 2006)
Chloroform (CHCl_3_)
Methanol (MeOH)
Ethyl acetate (EtOAc)
Acetone
Acetic acid (AcOH)
H_2_O, distilled
50 mM potassium carbonate in anhydrous methanol (K_2_CO_3_/MeOH)
1‐Butanol (BuOH)
Anhydrous methanol
0.5 M aqueous hydrogen chloride (HCl) (Current Protocols, 2006)
30% aqueous ammonia (NH_3_), BAKER INSTRA‐ANALYZED for trace metal analysis (J.T.Baker, CAS: 1336‐21‐6)
Ethanol (EtOH)
Methylamine, extra pure, 40% (w/v) in H_2_O (MeNH_2_) (Acros Organics, CAS: 74‐89‐5)

500‐ml, 250‐ml, 100‐ml and 50‐ml round‐bottom flasks
Magnetic stir bar
Glass stopper
Magnetic stirring plate
Thin‐layer chromatography plates, silica gel, 60 F254 (Supelco, cat. no. 105554)
UV lamp
Wool
Glass columns, 50‐mm width
Cation exchange resin, Dowex 50WX2‐100 H^+^ form (Supelco, cat. no. 217441)
Pasteur pipette
Test tubes
Rotary evaporator
Lyophilizer
pH indicator paper
Filter funnel with sintered glass disc
Silica gel, 60‐Å pore size, 230 to 400 mesh particle size, 40‐ to 63‐µm particle size (Supelco, cat. no. 60737)

Additional reagents and equipment for TLC (Meyers & Meyers, 2008) and column chromatography (Meyers, 2001)

#### Synthesis of 5‐carboxyuridine (3a) and 5‐carboxycytidine (3b)

1 Weigh the trifluoromethylated nucleoside **2a**/**2b** (1.0 g, 3.2 mmol) and transfer it into a 250‐ml round‐bottom flask equipped with a magnetic stir bar.
2 Add 100 ml of 0.5 M aqueous NaOH and close the flask with a glass stopper.
3 Place the flask on a magnetic stirring plate and stir at 60°C for 4 hr (substrate **2a**) or 1 hr (substrate **2b**).
4 Cool the mixture to room temperature, open the flask carefully, and check the reaction progress by TLC using 9:1 (v/v) CHCl_3_/MeOH followed by 5:3:1:1 (v/v/v/v) EtOAc/acetone/AcOH/H_2_O as eluents.
  After developing the TLC plate in the first solvent system, check if the substrate is present (under a UV lamp). Then develop the TLC in the second solvent system to observe the product spot.
  The R_f_ of 5‐carboxyuridine (**3a**) is 0.36.
  The R_f_ of 5‐carboxycytidine (**3b**) is 0.38.
5 Dilute the reaction mixture with 30 ml H_2_O.
6 Place a small wad of wool above the closed stopcock in the glass column. Fill the column with H_2_O and add cation exchange resin.
  The resin should be washed with H_2_O before use.
7 Load the reaction mixture on the column using a Pasteur pipette and pass through cation exchange resin. Rinse the resin with H_2_O.
8 Collect fractions into test tubes and check the presence of nucleoside by TLC. Concentrate the fraction containing product **3a**/**3b** using a rotary evaporator and lyophilize.
9 Characterize **3a**/**3b** using ^1^H NMR, ^13^C NMR, HRMS, and infrared (IR) spectroscopy.
  5‐Carboxyuridine (**3a**): 90% yield (0.83 g) as a white solid. ^1^H NMR (700 MHz, D_2_O) δ (ppm): 9.08 (s, 1H, H6), 5.96 (d, 1H, J = 2.8 Hz, H1′), 4.41 (dd, 1H, J = 2.8 Hz, J = 5.1 Hz, H2′), 4.29 (dd, 1H, J = 5.1 Hz, J = 7.0 Hz, H3′), 4.22 (dt, 1H, J = 3.1 Hz, J = 7.1 Hz, H4′), 4.05 (dd, 1H, J = 2.8 Hz, J = 13.3 Hz, H5′), 3.89 (dd, 1H, J = 3.5 Hz, J = 12.6 Hz, H5″). ^13^C NMR (176 MHz, D_2_O) δ (ppm): 166.08 (COOH), 164.33 (C4), 150.43 (C2), 149.20 (C6), 103.22 (C5), 90.65 (C1′), 83.95 (C4′), 74.36 (C2′), 68.47 (C3′), 59.66 (C5′). HRMS (ESI) m/z calculated for C_10_H_11_N_2_O_8_ [M‐H]^‐^ 287.0515; found 287.0522. IR (ATR) cm^‐1^: 3354 (O‐H), 1712 (C = O).
  5‐Carboxycytidine (**3b**): 85% yield (0.78 g) as a white solid. ^1^H NMR (700 MHz, D_2_O) δ (ppm): 9.24 (s, 1H, H6), 5.95 (d, 1H, J = 2.8 Hz, H1′), 4.40 (dd, 1H, J = 2.8 Hz, J = 4.9 Hz, H2′), 4.28 (dd, 1H, J = 4.9 Hz, J = 7.7 Hz, H3′), 4.24 (dt, 1H, J = 3.5 Hz, J = 7.0 Hz, H4′), 4.07 (dd, 1H, J = 2.8 Hz, J = 13.3 Hz, H5′), 3.89 (dd, 1H, J = 3.5 Hz, J = 13.3 Hz, H5″). ^13^C NMR (176 MHz, D_2_O) δ (ppm): 169.82 (COOH), 161.65 (C4), 151.65 (C2), 151.45 (C6), 102.72 (C5), 93.44 (C1′), 86.63 (C4′), 76.92 (C2′), 71.11 (C3′), 62.33 (C5′). HRMS (ESI) m/z calculated for C_10_H_12_N_3_O_7_ [M−H]^‒^ 286.0675; found 286.0684. IR (ATR) cm^‐1^: 3226 (O‐H), 1726 (C = O).

#### Synthesis of 5‐methoxycarbonyluridine (4a) and 5‐methoxycarbonylcytidine (4b) via orthoester intermediate (7a/7b)

**Figure 3:**
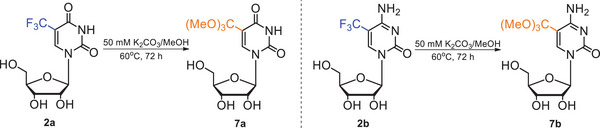
Synthesis of the orthoester derivatives, *5*‐(trimethoxymethyl)uridine (**7a**) and 5‐(trimethoxymethyl)cytidine (**7b**), intermediates in the synthesis of 5‐methoxycarbonyluridine/cytidine (**4a**/**4b**).

10 Weigh the trifluoromethylated nucleoside **2a**/**2b** (1.0 g, 3.2 mmol) and transfer it into a 250‐ml round‐bottom flask equipped with a magnetic stir bar.
11 Add 140 ml of 50 mM K_2_CO_3_/MeOH and close the flask with a glass stopper.
12 Place the flask on a magnetic stirring plate and stir at 60°C for 72 hr.
13 Cool the mixture to room temperature, open the flask carefully, and check the reaction progress by TLC using 4:1 (v/v) CHCl_3_/MeOH or 5:1:4 (v/v/v) BuOH/AcOH/H_2_O as development solvents.
  TLC analysis shows complete substrate consumption **2a**/**2b** and the presence of one product, identified as an orthoester derivative: 5‐(trimethoxymethyl)uridine (**7a**) or 5‐(trimethoxymethyl)cytidine (**7b**) (Fig. 3). If you wish to isolate **7a/7b**, evaporate the reaction mixture on a rotary evaporator and then purify by silica gel column chromatography using a gradient of MeOH in CHCl_3_ as eluent (0% to 15% for compound **7a**, 0% to 20% for compound **7b**). Characterize the compounds by ^1^H NMR. Otherwise, go to step 14 without purification.
  5‐(Trimethoxymethyl)uridine (**7a**): R_f_ = 0.33 (BuOH/AcOH/H_2_O, 5:1:4, v/v/v). ^1^H NMR (700 MHz, D_2_O) δ (ppm): 8.50 (s, 1H, H6), 5.94 (d, 1H, J = 2.8 Hz, H1′), 4.37 (dd, 1H, J = 2.8 Hz, J = 4.9 Hz, H2′), 4.26 (dd, 1H, J = 4.9 Hz, J = 7.0 Hz, H3′), 4.19‐4.16 (m, 1H, H4′), 4.01 (dd, 1H, J = 2.8 Hz, J = 12.6 Hz, H5′), 3.86 (dd, 1H, J = 3.5 Hz, J = 12.6 Hz, H5″), 3.21 (s, 9H, 3× O‐CH_3_).
  5‐(Trimethoxymethyl)cytidine (**7b**): R_f_ = 0.43 (CHCl_3_/MeOH, 4:1, v/v). ^1^H NMR (700 MHz, D_2_O) δ (ppm): 8.51 (s, 1H, H6), 5.92 (d, 1H, J = 2.8 Hz, H1′), 4.31 (dd, 1H, J = 2.8 Hz, J = 4.9 Hz, H2′), 4.24 (dd, 1H, J = 4.9 Hz, J = 7.7 Hz, H3′), 4.19‐4.16 (m, 1H, H4′), 4.04 (dd, 1H, J = 2.8 Hz, J = 12.6 Hz, H5′), 3.88‐3.84 (m, 1H, H5″), 3.23 (s, 9H, 3× O‐CH_3_).

#### Hydrolysis of orthoester derivative 7a/7b

14 For **7a**:
  - Dilute the mixture with anhydrous 30 ml MeOH.
  - Add cation exchange resin (Dowex H^+^) to the round‐bottom flask containing **7a** until the reaction is acidic (use a pH indicator paper).
  - Check the hydrolysis progress by TLC.
  - Filter off the resin (after ∼30 min) using a filter funnel and collect the solvent in a clean 500‐ml round‐bottom flask.
  - Rinse Dowex‐H^+^ with anhydrous MeOH.
    The Dowex‐H^+^ resin should be washed with H_2_O and anhydrous MeOH before use.
15 For **7b**:
  - Add 0.5 M aqueous HCl dropwise to the round‐bottom flask containing compound **7b** until the reaction is acidic (use a pH indicator paper).
  - Place the flask on a magnetic stirring plate and stir for 12 hr at room temperature.
  - Check the hydrolysis progress by TLC.
    Compound **7b** is stable under Dowex‐H^+^ acidification conditions, therefore procedure involves HCl.
16 Remove the solvent using a rotary evaporator under reduced pressure.
17 Purify the crude product **4a**/**4b** by silica gel column chromatography using a gradient of MeOH in CHCl_3_ as eluent (0% to 15% for nucleoside **4a**, 0% to 20% for nucleoside **4b**).
  The crude products are dry loaded onto the columns as described in Basic Protocol 1 (step 8).
18 Concentrate collected fractions under reduced pressure and lyophilize.
19 Characterize **4a**/**4b** using ^1^H NMR, ^13^C NMR, TLC, HRMS, and IR spectroscopy.
  5‐Methoxycarbonyluridine (**4a**): yield 90% (0.87 g) as a white solid. R_f_ = 0.2 (BuOH/AcOH/H_2_O, 5:1:4, v/v/v). ^1^H NMR (700 MHz, D_2_O) δ (ppm): 9.10 (s, 1H, H6), 5.96 (d, 1H, J = 2.5 Hz, H1′), 4.40‐4.39 (m, 1H, H2′), 4.31‐4.29 (m, 1H, H3′), 4.22‐ 4.20 (m, 1H, H4′), 4.07 (dd, 1H, J = 2.8 Hz, J = 13.0 Hz, H5′), 3.90 (dd, 1H, J = 3.4 Hz, J = 13.0 Hz, H5″), 3.88 (s, 3H, O‐CH_3_). ^13^C NMR (176 MHz, D_2_O) δ (ppm): 164.73 (O‐C = O), 162.15 (C4), 150.61 (C2), 149.13 (C6), 104.17 (C5), 90.58 (C1′), 83.78 (C4′), 74.39 (C2′), 68.35 (C3′), 59.48 (C5′), 52.43 (O‐CH_3_). HRMS (ESI) m/z calculated for C_11_H_13_N_2_O_8_ [M–H]^‒^ 301.0672; found 301.0680. IR (ATR) cm^‐1^: 2946 (C‐H), 1681 (C = O), 1087 (C‐O‐C).
  5‐Methoxycarbonylcytidine (**4b**): yield 72% (0.70 g) as a white solid. R_f_ = 0.38 (CHCl_3_/MeOH, 4:1, v/v). ^1^H NMR (700 MHz, D_2_O) δ (ppm): 9.21 (s, 1H, H6), 5.91 (bs, 1H, H1′), 4.33‐4.31 (m, 1H, H2′), 4.27‐4.24 (m, 1H, H3′), 4.20‐4.18 (m, 1H, H4′), 4.10‐4.08 (m, 1H, H5′), 3.91‐3.88 (m, 4H, O‐CH_3_, H5″). ^13^C NMR (176 MHz, D_2_O) δ (ppm): 165.77 (O‐C = O), 162.53 (C4), 154.33 (C2), 149.32 (C6), 96.90 (C5), 91.25 (C1′), 83.24 (C4′), 74.53 (C2′), 67.79 (C3′), 59.03 (C5′), 52.52 (O‐CH_3_). HRMS (ESI) m/z calculated for C_11_H_14_N_3_O_7_ [M‐H]^‐^ 300.0832; found 300.0842. IR (ATR) cm^‐1^: 2940 (C‐H), 1710 (C = O), 1098 (C‐O‐C).

#### Synthesis of 5‐cyanouridine (5a) and 5‐cyanocytidine (5b)

20 Weigh the trifluoromethylated nucleoside **2a**/**2b** (1.0 g, 3.2 mmol) and transfer it into a 250‐ml round‐bottom flask equipped with a magnetic stir bar.
21 Add 140 ml of 3:1 (v/v) 30% aqueous NH_3_/EtOH and close the flask with a glass stopper.
22 Place the flask on a magnetic stirring plate and stir at 60°C for 4 hr (substrate **2a**) or 3 hr (substrate **2b**).
23 Cool the mixture to room temperature, open the flask carefully, and check the reaction progress by TLC using 0.5:1:1:6 (v/v/v/v) H_2_O/EtOH/acetone/EtOAc as development solvents.
  The R_f_ of 5‐cyanouridine (**5a**) is 0.78.
  The R_f_ of 5‐cyanocytidine (**5b**) is 0.47.
24 Remove volatile components of the mixture on a rotary evaporator.
25 Purify the crude product **5a**/**5b** by silica gel column chromatography using a gradient of MeOH in CHCl_3_ as eluent (0% to 10% for nucleoside **5a**, 0% to 12% for nucleoside **5b**).
  The crude products are dry loaded onto the columns as described in Basic Protocol 1 (step 8).
26 Concentrate collected fractions under reduced pressure and lyophilize.
27 Characterize **5a**/**5b** using ^1^H NMR, ^13^C NMR, HRMS, and IR spectroscopy.
  5‐Cyanouridine (**5a**): yield 70% (0.60 g) as a white foam. ^1^H NMR (700 MHz, D_2_O) δ (ppm): 8.83 (s, 1H, H6), 5.89 (d, 1H, J = 2.8 Hz, H1′), 4.37 (dd, 1H, J = 2.8 Hz, J = 4.9 Hz, H2′), 4.24 (dd, 1H, J = 5.6 Hz, J = 7.0 Hz, H3′), 4.21‐4.19 (m, 1H, H4′), 4.03 (dd, 1H, J = 2.1 Hz, J = 12.6 Hz, H5′), 3.88 (dd, 1H, J = 3.5 Hz, J = 12.6, H5″). ^13^C NMR (176 MHz, D_2_O) δ (ppm): 162.40 (C4), 150.37 (C6), 150.09 (C2), 113.91 (CN), 90.77 (C1′), 89.01 (C5), 84.08 (C4′), 74.27 (C2′), 68.48 (C3′), 59.90 (C5′). HRMS (ESI) m/z calculated for C_10_H_10_N_3_O_6_ [M‐H]^‒^ 268.0570; found 268.0579. IR (ATR) cm^‐1^: 2241 (C≡N).
  5‐Cyanocytidine (**5b**): yield 70% (0.60 g) as a white foam. ^1^H NMR (700 MHz, D_2_O) δ (ppm): 8.78 (s, 1H, H6), 5.86 (d, 1H, J = 2.1 Hz, H1′), 4.32‐4.31 (m, 1H, H2′), 4.21‐4.20 (m, 2H, H3′, H4′), 4.06 (dd, 1H, J = 2.1 Hz, J = 13.3 Hz, H5′), 3.89 (dd, 1H, J = 3.5 Hz, J = 13.3 Hz, H5″). ^13^C NMR (176 MHz, D_2_O) δ (ppm): 163.31 (C4), 155.24 (C2), 150.66 (C6), 114.26 (CN), 91.45 (C1′), 83.61 (C4′), 81.17 (C5), 74.47 (C2′), 68.26 (C3′), 59.84 (C5′). HRMS (ESI) m/z calculated for C_10_H_11_N_4_O_5_ [M‐H]^‒^ 267.0729; found 267.0724. IR (ATR) cm^‐1^: 2225 (C≡N).

#### Synthesis of 5‐(N,N’‐dimethylamidinyl)uridine (6a)

28 Transfer 0.15 g (0.48 mmol) of nucleoside **2a** into a 50‐ml round‐bottom flask equipped with a magnetic stir bar.
29 Add 21 ml of 40% aqueous MeNH_2_ solution and close the flask with a glass stopper.
  Methylamine is a highly volatile compound with an irritating smell. This step should be performed under a well‐ventilated fume hood.
30 Place the flask on a magnetic stirring plate and stir at 60°C for 3 hr.
31 Cool the mixture to room temperature, open the flask carefully, and check the reaction progress by TLC using 5:1:4 (v/v/v) BuOH/AcOH/H_2_O as development solvents.
  The R_f_ of compound **6a** is 0.13.
32 Remove volatile components of the mixture on a rotary evaporator.
33 Purify the crude product **6a** by silica gel column chromatography using the mixture of CHCl_3_/MeOH/H_2_O (65:25:4, v/v/v) as eluent.
34 Concentrate collected fraction under reduced pressure and lyophilize.
35 Characterize **6a** using ^1^H NMR, ^13^C NMR, HRMS, and IR spectroscopy.
  5‐(N,N’‐Dimethylamidinyl)uridine (**6a**): 82% yield (124 mg). ^1^H NMR (700 MHz, D_2_O) δ (ppm): 8.46 (s, 1H, H6), 5.92 (d, 1H, J = 2.8 Hz, H1′), 4.36 (dd, 1H, J = 2.8 Hz, J = 4.9 Hz, H2′), 4.27‐4.25 (m, 1H, H3′), 4.20‐4.16 (m, 1H, H4′), 4.00 (dd, 1H, J = 2.8 Hz, J = 13.3 Hz, H5′), 3.85 (dd, 1H, J = 2.8 Hz, J = 13.0 Hz, H5″), 3.02 (s, 3H, C = N‐CH_3_), 2,99 (s, 3H, NH‐CH_3_). ^13^C NMR (176 MHz, D_2_O) δ (ppm): 160.18 (NH‐C = N), 155.60 (C4), 148.29 (C2), 144.22 (C6), 103.74 (C5), 90.49 (C1′), 83.80 (C4′), 74.29 (C2′), 68.56 (C3′), 59.91 (C5′), 30.91 (C = N‐CH_3_), 28.63 (NH‐CH_3_). HRMS (ESI) m/z calculated for C_12_H_17_N_4_O_6_ [M‐H]^‐^ 313.1148; found 313.1153. IR (ATR) cm^‐1^: 3201 (N‐H), 2925 (C‐H), 1644 (C = N).

#### Synthesis of 5‐N‐methylcarbamoylcytidine (6b)

36 Transfer 1.0 g (3.2 mmol) of nucleoside **2b** into a 250‐ml round‐bottom flask equipped with a magnetic stir bar.
37 Add 140 ml of 4% aqueous MeNH_2_ solution and close the flask with a glass stopper.
  To prepare this solution, use 40% aqueous MeNH_2_ and dilute it.
38 Place the flask on a magnetic stirring plate and stir at 60°C for 20 hr.
39 Cool the mixture to room temperature, open the flask carefully, and check the reaction progress by TLC using 5:1:4 (v/v/v) BuOH/AcOH/H_2_O as development solvents.
  The R_f_ of compound **6b** is 0.36.
40 Remove volatile components of the mixture on a rotary evaporator.
41 Purify the crude product **6b** by silica gel column chromatography using a gradient of 0% to 30% MeOH in CHCl_3_ as eluent.
  The crude product is dry loaded onto the column as described in Basic Protocol 1 (step 8).
42 Concentrate collected fraction under reduced pressure and lyophilize.
43 Characterize **6a** using ^1^H NMR, ^13^C NMR, HRMS, and IR spectroscopy.
  5‐N‐methylcarbamoylcytidine (**6b**): 65% yield (0.63 g). ^1^H NMR (700 MHz, D_2_O) δ (ppm): 8.60 (s, 1H, H6), 5.88 (d, 1H, J = 2.1 Hz, H1′), 4.33 (dd, 1H, J = 2.8 Hz, J = 5.6 Hz, H2′), 4.27 (dd, 1H, J = 4.9 Hz, J = 7.7 Hz, H3′), 4.20‐4.18 (m, 1H, H4′), 4.06 (dd, 1H, J = 2.8 Hz, J = 12.6 Hz, H5′), 3.89 (dd, 1H, J = 3.5 Hz, J = 12.6 Hz, H5″), 2.87 (s, 3H, CH_3_‐NH). ^13^C NMR (176 MHz, D_2_O) δ (ppm): 167.14 (CO‐NH), 163.55 (C4), 156.10 (C2), 143.70 (C6), 101.44 (C5), 90.95 (C1′), 83.48 (C4′), 74.37 (C2′), 68.19 (C3′), 59.76 (C5′), 26.04 (CH_3_‐NH). HRMS (ESI) m/z calculated for C_11_H_15_N_4_O_6_ [M‐H]^‐^ 299.0992; found 299.0998. IR (ATR) cm^‐1^: 3313 (N‐H), 2936 (C‐H), 1680 (C = O).

## COMMENTARY

### Background Information

The synthetic methods described in the literature for incorporating a ‘carbon substituent’ into the C5 position of uridine or cytidine include: hydroxymethylation (Dai et al., 2011; Ikeda et al., 1975; Sajiki et al., 2002); aminomethylation via the Mannich reaction (Jones et al., 1982; Reese & Sanghvi, 1984; Sekine et al., 1987); palladium‐catalyzed reactions of 5‐iodo‐nucleosides to introduce alkyl, alkenyl, or aryl groups (Crouch & Eaton, 1994; Karino et al., 2001; Liang et al., 2014); reaction of a C5‐lithiated nucleoside with an appropriate electrophile (Fu et al., 2010; Nawrot & Malkiewicz, 1989; Tanaka et al., 1986); substitution of 5‐halo derivatives with the generated carboanion (Borowski et al., 2019; Isenberg & Heidelberger, 1967); and radical malonylation induced by Mn(III) or Ce(IV) compounds (Kim et al., 1995).

All the above‐mentioned strategies require prior protection of the hydroxyl functions of ribose, as well as the selection of proper deprotection conditions to obtain the final product, making these methods costly and time‐consuming. The choice of the method for C5‐functionalization depends on the type of nucleobase (uracil or cytosine), the chemical status of the C5‐modifying group, and demand for regioselectivity. It is also crucial to consider the overall efficiency and scalability of the chosen synthetic route. Therefore, novel methodologies of 5‐substituted pyrimidine nucleosides should be straightforward and effective.

In this context, we paid attention to the CF_3_ group linked to the C5 carbon of uridine or cytidine, since it was expected to be easily convertible under alkaline conditions. The most common method for the regioselective installation of the CF_3_ group in heteroaromatic systems is the radical trifluoromethylation developed by Ji et al. (2011). It is a transition metal‐free approach based on easy‐to‐handle, commercially available reagents: CF_3_SO_2_Na (known as the Langlois reagent) and *tert*‐butyl hydroperoxide as the radical source. This method was successfully applied to regioselective monotrifluoromethylation of 2’‐deoxy nucleosides (Ito et al., 2017; Musumeci et al., 2013). In case of 5‐trifluoromethyluridine and 5‐trifluoromethylcytidine, two general methods based on peroxide‐generated CF_3_ radicals have been reported in the literature. The first method involves trifluoromethylation of U/C with gaseous CF_3_I in the presence of the Fenton oxidation reagent (Fe^2+/^H_2_O_2_/H_2_SO_4_; Y = 11% to 53%) (Uraguchi et al., 2008; Yamakawa et al., 2011; Yamakawa et al., 2015). The second method utilizes a photoinduced reaction with, for example, trifluoromethyl sulfones or the Langlois reagent as the source of CF_3_ radicals (Y = 38% to 42%) (Ghosh et al., 2019; Li et al., 2016; Liu et al., 2017).

The procedures described here allow the synthesis of C5‐substituted pyrimidine ribonucleosides in two stages: C5‐trifluoromethylation of U/C, followed by ‐CF_3_ conversion with oxygen or nitrogen nucleophiles at elevated temperatures, without the need to temporarily block the hydroxyl groups of the sugar residue. Based on this strategy, we elaborated the gram‐scale chemical synthesis of eight C5‐functionalized pyrimidine nucleosides containing the C5‐C ‘carbon substituent’ in good yields. The developed method serves as an attractive alternative to existing approaches.

### Critical Parameters and Troubleshooting

The described procedures should be carried out by an organic chemist familiar with basic chemical laboratory techniques, including evaporation, thin‐layer chromatography, and column chromatography. Characterization of compounds requires knowledge of ^1^H, ^13^C, and ^19^F NMR, HRMS, and IR spectroscopy. Operations that require the use of anhydrous solvents are indicated in the protocols. High‐purity reagents should be employed.

The efficiency of the CF_3_ group conversions depends on the purity of trifluoromethylated nucleosides **2a** and **2b**, synthesized according to Basic Protocol 1. The attempts to improve the conversion of C/U to 5‐trifluomethylated products **2a**/**2b** by increasing the excess of reagents (CF_3_SO_2_Na and TBHP) or extending the reaction time to 24 hr are ineffective and result in the formation of more by‐products, making the purification step more difficult. Double purification may be necessary to obtain CF_3_‐nucleosides of the desired quality.

### Understanding Results

The protocols described in this article represent optimized reaction conditions. By adhering to the provided procedures, it is expected that comparable results can be achieved (see Table 1). However, it should be noted that the final yields of the isolated compounds may differ from those presented, primarily influenced by the quality of reagents, especially the Langlois reagent (Basic Protocol 1), and the effectiveness of chromatographic purification.

**Table 1:** Summary of 5‐CF_3_U (**2a**) and 5‐CF_3_C (**2b**) Conversions

Substrate	Reagent	Time (h)	Product	Yield (%)
2a	0.5 M aqueous NaOH	4	3a	90
2b	0.5 M aqueous NaOH	1	3b	85
2a	(1) 50 mM K_2_CO_3_/MeOH (2) Dowex H^+^	72	4a	90
2b	(1) 50 mM K_2_CO_3_/MeOH (2) 0.5 M aqueous HCl	72	4b	72
2a	3:1 (v/v) 30% aqueous NH_3_/EtOH	4	5a	70
2b	3:1 (v/v) 30% aqueous NH_3_/EtOH	3	5b	70
2a	40% aqueous MeNH_2_	3	6a	82
2b	4% aqueous MeNH_2_	20	6b	65

### Time Considerations

The synthesis and purification of trifluoromethylated nucleosides **2a** and **2b**, according to Basic Protocol 1, takes 3 to 4 days. An additional purification step (if necessary) can delay the work. Basic Protocol 2 requires 3 to 4 weeks.

### Author Contributions

**Karolina Podskoczyj**: Investigation; methodology; validation; writing original draft. **Anna Kuszczynska**: Investigation. **Agnieszka Dziergowska**: Investigation. **Grazyna Leszczynska**: Conceptualization; methodology; supervision; writing review and editing.

### Conflict of Interest

The authors declare no conflict of interest.

## Data Availability

Data sharing not applicable; no new data generated.
